# Bridging the gap: the role of student conferences in advancing sport and exercise medicine in the medical school curricula

**DOI:** 10.1186/s12909-026-08678-x

**Published:** 2026-01-28

**Authors:** Yunus Ali, George Monaghan, Allegra Wisking, Demi Ojo, Iris Zieler, Eeshaan Ghanekar, Courtney Kipps

**Affiliations:** 1 University College London Medical School, London, UK; 2 Division of Surgery and Interventional Sciences, University College London, London, UK; 3 NHS Greater Glasgow and Clyde, Glasgow, UK; 4 NHS Leeds Teaching Hospitals Trust, Leeds, UK; 5 Bedfordshire Hospitals NHS Foundation Trust, Bedford, UK; 6 Surrey and Sussex Healthcare NHS Trust, Surrey, UK

**Keywords:** Medical education, Barriers, Student-led, Conference, Sport and Exercise Medicine, Under-representation, Medical School, Career Pathways

## Abstract

**Background:**

Sport and Exercise Medicine (SEM) is an expanding specialty yet remains under-represented in medical school curricula. Persistent barriers limit medical students’ understanding of SEM and awareness of career pathways. Student-led conferences may address these gaps by providing targeted exposure and early engagement. This study evaluated the impact of a national, student-led SEM conference on delegates’ knowledge, preparedness, and perceptions of SEM across UK medical schools.

**Methods:**

The 2024 National Undergraduate SEM Conference was hosted by University College London and the Institute of Sport, Exercise and Health. Seventy-three delegates from sixteen UK medical schools completed pre- and post-conference questionnaires. Responses were rated on a 5-point Likert scale and analysed using Wilcoxon signed-rank tests. Free-text data was collected and underwent thematic analysis.

**Results:**

Significant improvements (*p* < 0.0001) were seen in awareness of SEM career pathways (+ 55.7%), preparedness for a SEM career (+ 46.2%), understanding of SEM (+ 44.4%), and awareness of barriers to accessing careers in SEM (+ 41.4%). Interest in SEM also increased (*p* = 0.0034). 86% of delegates wanted more SEM-related opportunities in medical school; 87% felt current SEM curriculum coverage was inadequate; and 70% felt SEM to be under-supported by medical schools in content exposure and opportunity. Qualitative themes highlighted the need for greater clinical exposure, mentoring, and student-led opportunities.

**Conclusion:**

A one-day, national, student-led SEM conference significantly improved delegates’ understanding, sense of preparedness, and career awareness, while identifying curriculum deficiencies. Through the outcomes of its practical workshops and lectures, and its examination of systemic barriers to entry, this national conference extends prior single-centre findings. Student-led initiatives offer a scalable, low-cost approach for enhancing SEM education of medical students by supplementing educational gaps in the medical school curriculum.

**Clinical trial number:**

Not Applicable.

**Supplementary Information:**

The online version contains supplementary material available at 10.1186/s12909-026-08678-x.

## Background

Sport and Exercise Medicine (SEM) is defined by the Faculty of Sport and Exercise Medicine UK as a ‘dynamic medical specialty that employs a multidisciplinary approach to address issues preventing individuals from initiating or resuming physical activity’ [1]. It encompasses the prevention, diagnosis, treatment, and rehabilitation of injuries and illnesses related to physical activity, while also promoting exercise for general health and chronic disease management.

SEM is an emerging and expanding specialty, increasingly recognised for its value in managing musculoskeletal (MSK) conditions, supporting rehabilitation, and contributing to public health through physical activity promotion. MSK conditions are common in the UK, affecting nearly 30% of the population (approximately 18.8 million individuals) [2]. They account for 1 in 7 primary care consultations, with many patients presenting multiple times due to unresolved or chronic symptoms [3]. The high prevalence of MSK cases in general practice has led to the need for improved medical training to reduce rates of repeat consultations [4]. Beyond MSK care, SEM is integral to the management and prevention of chronic conditions such as type 2 diabetes, cardiovascular disease, and obesity, conditions in which physical inactivity is a key risk factor. SEM interventions aim to improve functional capacity, reduce disease burden, and consequently lower healthcare costs [5]. The Office for Health Improvement and Disparities (OHID) estimates that 1 in 6 deaths in the UK are linked to physical inactivity, costing the UK approximately £7.4 billion annually [6]. The current UK Chief Medical Officers’ (CMO) Guidelines for physical activity recommend that each week adults should partake in at least 150 min of moderate intensity activity (e.g. brisk walking, cycling), or 75 min of vigorous intensity activity (e.g. running), or even shorter durations of very vigorous intensity activity (e.g. sprinting, stair climbing) alongside muscle strengthening on at least 2 days a week [7]. Data from the 2022 Health Survey for England indicate that approximately a third of adults are failing to meet the CMO’s guidelines, further highlighting the need for initiatives that educate the population on the importance of exercise [8].

Despite its clinical relevance, significant challenges to pursue a career in SEM remain, both at undergraduate and postgraduate levels. While interest in SEM among medical students and foundation doctors is evident - a recent survey distributed to medical students and foundation doctors between October 2022 and January 2024 found that 22.4% of 144 respondents were considering a career in SEM [9] - opportunities to explore the specialty are limited, and SEM-specific career guidance is lacking. Notably, only 43.7% of respondents were aware of the existence of SEM specialty training, and only 29.2% knew that SEM doctors exist within the NHS [9]. This issue is compounded by the limited presence of SEM content in many UK medical school curricula. The General Medical Council’s *Outcomes for Graduates* 2018 document outlines a set of high-level principles from which medical school curricula are derived [10]. It defines the knowledge, skills, and behaviours expected of medical graduates, but it does not mandate the teaching of specific medical specialties. While this affords medical schools discretion in how disciplines are mapped and delivered, it can contribute to variability in specialty coverage and emphasis, particularly for smaller or emerging specialties, such as SEM. This highlights the importance of medical schools explicitly signposting a comprehensive and up-to-date range of clinical specialties within their curricula.

Currently, SEM teaching in the UK averages 4.5 h over the 5 years of medical school [11]. Medical schools traditionally provide a minimum of 5500 h of teaching (as advised by the European Directive 2005/36/EC), thus making SEM teaching approximately < 0.1% of its contents [12]. The assessment and management of MSK conditions are key competencies of the SEM clinician, requiring knowledge and skills that overlap with other MSK related specialties (Trauma and Orthopaedics, Rheumatolgy etc.) [13] Medical students and resident doctors frequently report a lack of confidence in their knowledge of MSK medicine [14, 15]; a 2018 study found that approximately half of graduating medical students in the UK felt confident in giving accurate advice on physical activity [16]. Objective assessment of MSK competence has yielded similar results. In 1998, Freedman and Bernstein introduced a validated examination to assess knowledge and competency in MSK medicine [17]. A 2015 study of 210 graduating UK medical students found that despite 40% of them feeling subjectively competent in MSK medicine, only 21% of them passed Freedman and Bernstein’s MSK examination [18]. These findings reinforce the lack of progress and reform in MSK and SEM education despite its societal relevance.

Student-led conferences have emerged as a means of addressing educational gaps during medical school. These initiatives can provide targeted exposure to underrepresented specialties, enhance understanding of career pathways, and help build student confidence in pursuing specialties not widely covered in the undergraduate curriculum. This has been repeatedly demonstrated by studies using pre- and post-conference questionnaires, which have observed increases in self-reported knowledge of surgical specialties following surgical conference attendance (*p* < 0.001) [19, 20]. A study of a one-day, student-led SEM conference at King’s College London, found significant improvements in students’ self-reported understanding of SEM, in addition to increased confidence and interest in pursuing a career in the specialty [21]. Importantly, these outcomes were achieved without curriculum change or institutional intervention, underscoring the potential of peer-led, low-cost approaches to address gaps in medical education. Building upon these findings, in addition to keynote lectures, our national conference incorporated practical workshops, reflecting the importance of hands-on experience in SEM and potentially contributing to improved learning outcomes. Furthermore, unlike that of Dadrewalla et al., this study identified barriers to entry into SEM, providing actionable insights that may inform future educational strategies and policy change. Evaluation of this conference aimed to:

- Review the availability and accessibility of SEM teaching in medical school curricula, benchmarking against national educational standards (e.g. GMC *Outcomes for Graduates*, BASEM guidelines).
- Evaluate the impact of a student-led SEM conference on attendees’ understanding of SEM as a career option.
- Assess changes in student understanding of the role of SEM as a medical specialty, including perceived barriers to access and potential patient benefits, following conference participation.

By analysing pre- and post-conference questionnaire responses, this audit explores how structured, student-focused initiatives can raise awareness, improve perceptions, and encourage consideration of SEM as a viable and impactful career pathway.

## Methods

### Study design

A before-and-after study design using pre- and post-conference questionnaires was employed to evaluate the impact of the National Undergraduate Sports and Exercise Medicine Society (USEMS) Conference 2024 on attendees’ knowledge, perceptions, and attitudes towards SEM as a specialty. The study integrated both quantitative and qualitative elements.

### Conference overview

**Fig. 1:**
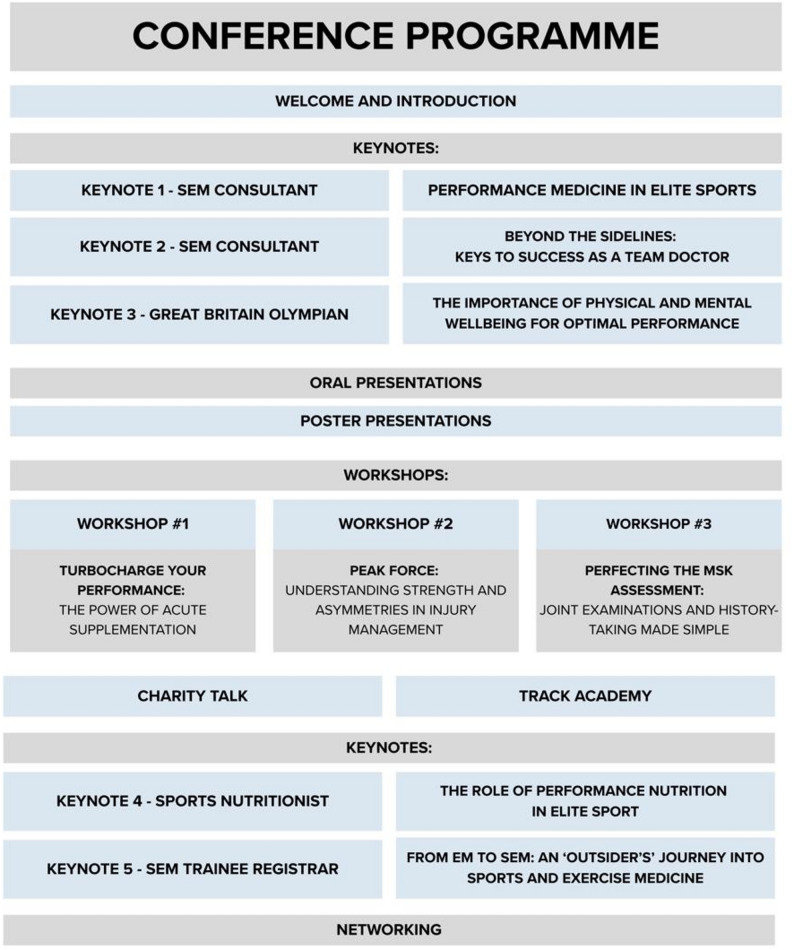
Conference Programme

The conference was held in November 2024 at University College London (UCL) and the Institute of Sport, Exercise and Health (ISEH), organised by UCL Sports and Exercise Medicine Society on behalf of the Undergraduate Sports and Exercise Medicine Society (USEMS). The event featured keynote presentations from SEM consultants and registrars, a Great Britain Olympian and a Sports Nutritionist, alongside practical workshops. The workshops were led by ISEH-affiliated clinicians and scientists and focused on MSK history-taking, use of supplementation in sport, and understanding strength and asymmetries in injury management using performance metrics. The programme also included oral and poster presentations, showcasing delegate research, and structured networking sessions (Fig. 1).

### Participants

A total of 115 individuals attended the conference. Of these, 73 attendees met the inclusion criteria for this study: attendance at the conference, completion of both pre- and post-conference questionnaires in full, and provision of informed consent. Attendees were excluded from analysis if they did not meet all the aforementioned criteria. The final sample comprised 56 medical students, 9 BSc students, 2 physiotherapy students, 4 MSc students, and 2 medical doctors. Ages ranged from 18 to 29 years (mean age = 21.6), with 42 male and 31 female participants representing 16 UK medical schools (Table 1).

**Table 1:** Participant demographics

Characteristic	Value
Age, years (mean ± SD)	21.6 ± 2.3
Biological Sex, n (%)	Male 42 (58); Female 31 (42); Prefer not to say 0 (0)
Role, n (%)	Medical doctor 2 (3); Medical student 56 (77); BSc student 9 (12); Physiotherapy student 2 (3); MSc student 4 (5)
Medical schools represented (*n* = 16)	Cardiff University; Hull York Medical School; Imperial College London; King’s College London; Queen Mary University of London; University College London; University of Birmingham; University of Bristol; University of East Anglia; University of Exeter; University of Glasgow; University of Leeds; University of Leicester; University of Manchester; University of Nottingham; University of Sheffield

### Questionnaire

**Fig. 2:**
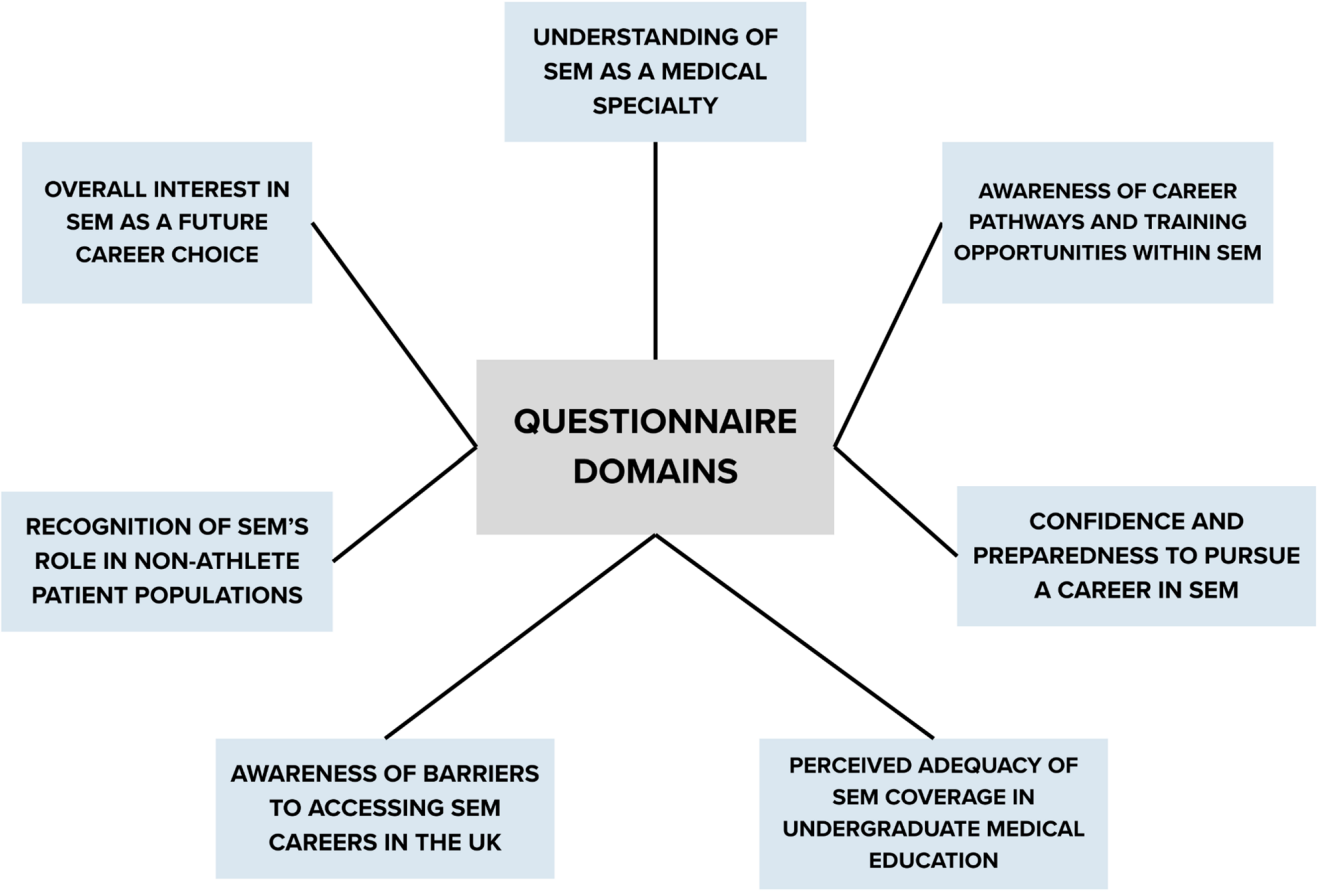
Questionnaire domains

The questionnaires were developed specifically for this study based on the educational objectives of the conference, however the question domains were derived from recurring themes found in the literature regarding barriers and gaps to SEM education [16, 22], as well as national competency guidance from BASEM and FSEM. A Likert-scale item was asked for each of the following domains (Fig. 2), along with some text-based questions, covering understanding, awareness, preparedness, perception of medical school coverage, recognition, barriers and interest in SEM (see appendix).

Both pre and post questionnaires included seven identical 5-point Likert-scale items (1 = strongly disagree; 5 = strongly agree). Additionally, the pre-conference questionnaire included three Likert-scale items on perceived SEM coverage and opportunities within medical school, and two open-ended questions exploring perceived barriers and preparedness to pursue SEM and an additional open-ended question asking what delegates hoped to gain from attending the conference. The post-conference questionnaire also included three additional questions exploring what delegates enjoyed most about the conference, how the conference could be improved, and whether they would recommend it to others.(see appendix). Likert-scale items were chosen for their suitability in capturing changes in attitudes and perceptions over time, enabling comparison between pre- and post-conference responses, similar to other existing conference research [19, 23]. Free-text questions were included to allow participants to elaborate on their views, enabling deeper exploration of perceived barriers, motivations, and career aspirations within SEM.

Pre- and post-surveys were designed using Google Forms and distributed electronically. The pre-conference survey was sent via email to all registered ticket holders prior to the event. The post-conference survey was distributed at the end of the conference via QR codes and also at a later date via email. Where duplicate submissions were received, only the most recent response was retained for analysis. All data was anonymised prior to analysis.

### Ethical approval and consent to participate

Ethical exemption was granted by the UCL Research Ethics Committee as the project fulfilled criteria for service evaluation and educational audit. This study adhered to the principles outlined in the Declaration of Helsinki. All participants were informed of the study’s purpose, and participation was voluntary.

### Statistical analysis

Statistical analyses were conducted using Stata software (StataCorp., Texas, USA). Data are presented as mean ± standard deviation (SD). Normality was assessed using the Shapiro-Wilk test, which indicated non-parametric distribution of the data, justifying the use of the Wilcoxon signed-rank test to evaluate differences between pre- and post-conference questionnaire responses. All statistical tests were two-tailed, and all comparative analyses were conducted within a 95% confidence interval.

For questions related to perceptions of SEM teaching within undergraduate curricula, only responses from medical students and doctors were included in the analysis to reflect direct experience of medical school.

## Results

Statistically significant improvements were observed in all questionnaire domains. The largest relative improvement was in awareness of career pathways in SEM (+ 55.7%, Pre: 2.7 ± 0.9; Post: 4.1 ± 0.7), which was a primary aim of the conference. Notable increases were seen in participants’ self-reported preparedness for a career in SEM (+ 46.2%, Pre: 2.7 ± 0.9; Post: 4.0 ± 0.7), understanding of what a career in SEM entails (+ 44.4%, Pre: 2.9 ± 0.8; Post: 4.2 ± 0.6), and awareness of potential barriers (+ 41.4%, Pre: 2.9 ± 1.0; Post: 4.2 ± 0.8), all with P values < 0.0001.

While initial interest in SEM was already high (4.2/5), a statistically significant increase was still reported post-conference (4.4/5) (*P* = 0.0034) (Table 2). Participants were also invited to provide free-text responses, allowing them to suggest ways that could help them feel more prepared in pursuing a career in SEM (Table 3) and the barriers that they have faced (Table 4).

**Table 2:** Mean likert scale scores in both pre- and post-conference surveys

Question	Number of responses (*n*)	Pre-Conference Response (mean ± SD)	Post-Conference Response (mean ± SD)	Percentage Change (%)
I am prepared to be able to pursue a career in SEM	73	2.7 ± 0.9	4.0 ± 0.7	+ 46.2**
I understand what SEM entails as a specialty	73	2.9 ± 0.8	4.2 ± 0.6	+ 44.4**
I understand the different pathways into SEM	73	2.9 ± 0.9	4.2 ± 0.6	+ 44.2**
I know the different career paths available within SEM	73	2.7 ± 0.9	4.1 ± 0.7	+ 55.7**
I am aware of the barriers to accessing SEM services in the current healthcare system	73	2.9 ± 1.0	4.2 ± 0.8	+ 41.4**
I understand how SEM can benefit patient populations beyond athletes (e.g. elderly or those with chronic conditions)	73	4.0 ± 0.9	4.1 ± 0.9	+ 4.2%
I am interested in pursuing SEM as a future specialty	73	4.2 ± 0.6	4.4 ± 0.6	+ 6.6*

* = statistically significant in the p<0.01 interval

** = statistically significant in the p<0.0001 interval

**Table 3:** Free-text responses to the question: “What could make you feel more prepared for a career in SEM?”

Participant Responses	
“More clinical experience at medical school”	“More conferences like this one”
“More accessible ways to meet with, contact and learn from healthcare professionals that have gone pursued a SEM career”	“More support from those already within the specialty to advertise”
“More SEM teaching at medical school”	“More shadowing/mentoring opportunities at medical school”

**Table 4:** Free-text responses to the question: “Are there any barriers into SEM that you have encountered?”

Participant responses	
“Lack of teaching at medical school”	“(Lack of) placements shadowing professionals in the field”
“Lack of exposure on the medical school curriculum”	“Lack of education on how exactly to break into the field and what sort of jobs are available”
“I just think there still isn’t a huge amount of information available about SEM for medical students”	“Difficult to get placements.”

86% of participants indicated that they would like to see an increase in the number of SEM-related opportunities within the medical school curriculum. 70% of participants disagreed with the statement that SEM training is adequately supported in undergraduate medical education, and 87% disagreed that their medical school curriculum adequately addresses SEM (Table 5).

**Table 5:** Mean likert scale scores and average agreement levels of participant responses to domains regarding SEM opportunities, support and depth of coverage in medical school

Statement	Number of responses (*n*)	Pre-Conference Response (mean ± SD)	% of participants who disagree (Likert 1 or 2)	% of participants who neither agree nor disagree (Likert 3)	% of participants who disagree (Likert 1 or 2)
Training in SEM is adequately supported in undergraduate medical education.	73	2.1 ± 1.1	70	22	8
I would like more SEM-related opportunities in medical school curricula	73	4.5 ± 0.7	0	14	86
For medical students – I believe that my medical school curriculum covers SEM in adequate depth	62	1.9 ± 0.7	87	11	2

## Discussion

### Barriers to SEM and clinical implications

In this national SEM conference, pre-conference ratings for understanding of SEM pathways (mean 2.9 ± 0.9) and what the specialty entails (mean 2.9 ± 0.8) were low and the perceived curriculum coverage was limited (mean 1.9). Immediately after the conference, ratings improved across these domains (44% relative increase). These changes reflect self-reported gains in preparedness and awareness in a cohort that was predominantly composed of medical students (77%).

Limited exposure to SEM within medical school curricula is a primary barrier to access and understanding. Participants’ perceptions align with reports that SEM content in UK undergraduate curricula is minimal and inconsistently delivered [11, 24]. A recent report of clinical specialty training in UK undergraduate medical schools showed that, unlike other specialties such as surgery and psychiatry, SEM often lacks dedicated timetabled teaching and exposure to the specialty is typically gained through optional or elective modules [24].

Misconceptions persist that SEM is largely about “elite sport”. In reality, the specialty encompasses musculoskeletal care across the life course and the “exercise is medicine” principle is relevant to all clinicians [22]. Against this backdrop, our data suggests that a brief, targeted exposure, over a one-day, student-led event can improve outcomes such as perceived knowledge, confidence and awareness. This echoes findings from other student-led or short-format interventions in under-taught specialties (e.g., neurosurgery, oncology) and from prior SEM society conferences showing gains in self-reported understanding and interest [21, 23, 25–27].

Student-led initiatives, including conferences, workshops and society events, offer a practical means of improving the limited exposure to SEM in UK medical school curricula. The outcomes of our national conference mirror those of similar interventions, namely the aforementioned Dadrewalla et al. study [21], as well as an online survey conducted by the Royal College of General Practitioners (RCGP) [28] and reinforce that impactful learning experiences can be created outside the formal curriculum. Locally run, student-organised events can provide meaningful education and early career inspiration in SEM.

Wider workforce factors, such as the limited training numbers, and the relatively small specialist register, help contextualise why students may perceive the field as “niche” [29–31]. However, these system factors were not the object of our evaluation and are best treated as background, rather than as explanatory drivers of our measured outcomes.

Change is slow and constrained by competing demands, however the success of the conference demonstrated there is student interest in SEM. This could serve as supporting evidence to encourage medical schools to expand and enhance SEM teaching within the curriculum.

### Limitations

While our study offers significant findings to the body of SEM and medical education literature, several limitations must be acknowledged. The cohort completing the survey may not be representative of the wider UK medical student population, despite data being collected at a national conference. As the event targeted students interested in SEM, selection bias is likely, and the true level of awareness and understanding among the general student body may be lower than our results suggest. The scale and prominence of this conference may also have contributed to its impact: a national platform and high-profile speakers could enhance effects relative to smaller, local initiatives with fewer resources. As with all survey-based studies using Likert scales, the subjective nature of self-reported perceptions must be considered; improvements in perceived understanding or preparedness may not directly reflect objective knowledge or competence. Finally, although we observed consistency across domains and high statistical significance, our relatively small sample size may have inflated apparent effect sizes.

## Conclusion

This study demonstrates that a student-led conference can effectively raise awareness of SEM and contribute to improved understanding of this specialty within UK medical education. Statistically significant improvements in participants’ understanding of SEM career pathways, specialty scope and clinical relevance confirm that targeted, peer-organised interventions can enhance education in this area.

While student-led initiatives offer an immediate and scalable solution, they should not be viewed as a permanent substitute for formal curriculum integration. The success of this conference - drawing attendees from 16 medical schools and demonstrating measurable educational impact within a single day - highlights both the interest in SEM education and the feasibility of delivering focused educational content through peer-led formats. These findings support the need for clearer information and education relating to SEM as part of the MSK content of undergraduate medical curricula.

The educational impact achieved through this national conference demonstrates the potential to expand SEM awareness among interested medical students. Although the authors acknowledge the challenges of prioritising content with already crowded medical school curricula, our findings indicate a significant student interest in SEM, alongside a majority opinion that SEM is currently underrepresented. We hope this will open the floor for discussions regarding increasing undergraduate exposure to the specialty, moving beyond optional modules and elective placements, therefore better serving students’ understanding of career options within this field.

Sustainable improvement will likely require both grassroot student-led initiatives and institutional consideration of how this specialty is represented within medical curricula.

## Supplementary Information

Supplementary Material 1.

## Data Availability

All data supporting the findings of this study are included in this published article and its supplementary information files.

## Appendix

Appendix 1. Conference questionnaires (pre- and post-conference items)

Instructions: Please indicate your agreement with the following statements.

Likert response options: 1 = Strongly disagree; 2 = Disagree; 3 = Neither agree nor disagree; 4 = Agree; 5 = Strongly agree.

Consent item (included in both questionnaires; required):

I consent to my anonymised answers being stored and used for future research/quality improvement purposes. (Yes/No)

A Likert-scale items included in both pre- and post-conference questionnaires (5-point)
  - I am prepared to be able to pursue a career in SEM.
  - I understand what SEM entails as a specialty.
  - I understand the different pathways into SEM.
  - I know the different career paths available within SEM.
  - I am aware of the barriers to accessing SEM careers in the current healthcare system.
  - I understand how SEM can benefit patient populations beyond athletes (e.g., elderly or those with chronic conditions).
  - I am interested in pursuing SEM as a future specialty.
B Additional items included in the pre-conference questionnaire only
  Likert-scale (5-point)
  - Training in SEM is adequately supported in undergraduate medical education.
  - I would like to see more SEM-related opportunities in medical school curricula.
  - The medical school curriculum covers SEM in adequate depth.
  Open-ended (free text)
  - Are there any barriers into SEM that you have encountered?
  - What could make you feel more prepared for a career in SEM?
  - What do you hope to gain from attending this conference?

**Additional items included in the post-conference questionnaire only**

Open ended (free text)

- What aspects of the conference did you find the most useful/enjoyable?
- How could we improve this conference for future years?
- Would you recommend this conference to others?

## Abbreviations

BASEM: British Association of Sport, Exercise and Medicine
BSc: Bachelor of Science
BJSM: British Journal of Sports Medicine
CMO: Chief Medical Officer
FSEM: Faculty of Sport, Exercise and Medicine
GMC: General Medical Council
ISEH: Institute of Sport, Exercise and Health
MSc: Master of Science
MSK: Musculoskeletal
OHID: Office for Health Improvement and Disparities
SD: Standard deviation
SEM: Sport and Exercise Medicine
UCL: University College London
USEMS: Undergraduate Sports and Exercise Medicine Society
